# Deep learning and social network analysis elucidate drivers of HIV transmission in a high-incidence cohort of people who inject drugs

**DOI:** 10.1126/sciadv.abf0158

**Published:** 2022-10-19

**Authors:** Steven J. Clipman, Shruti H. Mehta, Shobha Mohapatra, Aylur K. Srikrishnan, Katie J. C. Zook, Priya Duggal, Shanmugam Saravanan, Paneerselvam Nandagopal, Muniratnam Suresh Kumar, Gregory M. Lucas, Carl A. Latkin, Sunil S. Solomon

**Affiliations:** ^1^ Division of Infectious Diseases, Department of Medicine, Johns Hopkins University School of Medicine, Baltimore, MD, USA.; ^2^ Department of Epidemiology, Johns Hopkins Bloomberg School of Public Health, Baltimore, MD, USA.; ^3^ YR Gaitonde Centre for AIDS Research and Education (YRGCARE), Chennai, India.; ^4^ Department of Health, Behavior and Society, Johns Hopkins Bloomberg School of Public Health, Baltimore, MD, USA.

## Abstract

Globally, people who inject drugs (PWID) experience some of the fastest-growing HIV epidemics. Network-based approaches represent a powerful tool for understanding and combating these epidemics; however, detailed social network studies are limited and pose analytical challenges. We collected longitudinal social (injection partners) and spatial (injection venues) network information from 2512 PWID in New Delhi, India. We leveraged network analysis and graph neural networks (GNNs) to uncover factors associated with HIV transmission and identify optimal intervention delivery points. Longitudinal HIV incidence was 21.3 per 100 person-years. Overlapping community detection using GNNs revealed seven communities, with HIV incidence concentrated within one community. The injection venue most strongly associated with incidence was found to overlap six of the seven communities, suggesting that an intervention deployed at this one location could reach the majority of the sample. These findings highlight the utility of network analysis and deep learning in HIV program design.

## Teaser

Social and spatial network structures are critical drivers of HIV incidence among people who inject drugs.

## INTRODUCTION

The recent severe acute respiratory syndrome coronavirus 2 (SARS-CoV-2) pandemic has highlighted challenges around controlling the spread of infectious diseases. At the most fundamental level, controlling outbreaks relies on breaking transmission chains, as infectious diseases largely propagate via connections of individuals, either directly via bodily fluids or indirectly through droplets and fomites (*1*). These connections and the underlying networks that they represent hold the key to understanding how diseases spread through a population and can lead to targeted intervention strategies to interrupt transmission (*2*, *3*).

Network-based intervention approaches can be particularly powerful for combating epidemics among populations that are linked by a common behavior (e.g., needle sharing) that may confer a greater risk of infections, such as HIV or hepatitis C virus (HCV) in people who inject drugs (PWID) (*4*–*7*). PWID in low- and middle-income countries (LMICs), in particular, account for some of the fastest-growing HIV epidemics globally (*8*–*10*); however, HIV transmission in real-world injection networks is still poorly understood, even less so in LMIC settings where HIV incidence and opioid use disorders are the highest (*11*–*14*). Given the limited resources available and the hard-to-reach nature of these populations, key questions around the implementation of network-based interventions for PWID typically relate to efficiency and the deployment of targeted interventions that can most effectively interrupt transmission in a population. The seminal Colorado Springs social network study led early investigation into the role of networks and indirect partner ties on HIV transmission among PWID (*15*). However, large longitudinal network studies among PWID are limited; a cohort of 388 PWID in Melbourne, Australia is currently one of the only longitudinal studies to date (*16*). This has consequently limited empirical evidence on the strength and potential for HIV transmission and, conversely, diffusion of interventions through injection partner networks (i.e., social networks of PWID where connections/ties represent persons with whom they inject drugs, regardless of needle/syringe sharing). This understanding can be critical to the design of network-based interventions. Furthermore, while some geospatial network studies have been conducted among sexual minorities (*17*–*20*), the role of spatial networks (e.g., injection venues) on HIV transmission among PWID has not been well described.

Because of the resource-intensive nature of enumerating complete networks of PWID, many network studies to date have been limited to static egocentric network designs, where an individual (index) provides information on their injection/sexual partners, but those partners (known as alters) are not recruited into the study. This is in contrast to a sociometric design where named partners are recruited into the study and, in turn, asked to name and recruit their partners, thereby capturing indirect ties (e.g., partner of a partner) of the initial index. The simpler egocentric designs, although most commonly used, are often poor representations of true network dynamics because real-world networks are typically much larger (considering alters of an index can have ties to alters of their own), overlap with one another, and contain substantial spatial and temporal components (*21*–*25*). Network-based interventions require a detailed understanding of these underlying network structures, extending beyond immediate ties, and incorporating spatial and temporal dynamics to target interventions efficiently. Network modeling studies of HIV sometimes take these larger network structures into account; however, models are still most often constructed using empirical data from egocentric studies with broader network properties inferred using methods such as exponential random graph models. Even in settings where more complete network data are available, they often lack detailed phenotype information or individual node characteristics that could represent significant drivers or risk factors for transmission.

Beyond the data collection challenges, the non-Euclidean nature of graphs has posed challenges for analyzing network data, and established methods such as spectral clustering have needed to discard available information or rely on summary measures of network centrality such as degree (number of edges node has), betweenness (extent to which node serves as a bridge between other nodes), or closeness centrality (proximity to other nodes in the network). Computational advancements in deep learning, specifically graph neural networks (GNNs), have made it possible to perform more powerful and complex analyses on network data, such as predicting links, network forecasting, or overlapping community detection (*26*, *27*). GNNs offer a novel opportunity to understand HIV transmission dynamics in ways that were not previously possible, and these deep learning approaches can allow network data to be harnessed more fully to identify optimal intervention approaches.

Here, we describe, what is to our knowledge, one of the first longitudinal sociometric and spatial network studies in a cohort of 2512 PWID from New Delhi, India. India is uniquely situated between the two largest heroin-producing regions in the world and is home to the largest number of opioid users globally (*12*). We present HIV incidence and the sociospatial dynamics in the cohort, and we leverage network analysis and deep learning methods to identify correlates of HIV infection and optimal points of intervention to efficiently interrupt HIV transmission in this population.

## RESULTS

### Recruitment and participant network characteristics

A total of 2502 PWID were recruited from 10 seeds who initiated recruitment in November 2017 (total *n* = 2512). Median age was 26 years [interquartile range (IQR): 22 to 34], and 2489 (99%) were male. The majority male population reflects the epidemiology of drug use in India and does not stem from any inherent recruitment bias (*28*–*31*). A total of 2504 (99%) participants reported injecting in the prior 6 months with an average of two injections per day, and 51% reported sharing injection paraphernalia in the 6 months before enrollment. The most commonly injected drugs were buprenorphine and heroin (Table 1). HIV prevalence at baseline was 37.0% (928 of 2506). Of those who tested positive for HIV antibodies, 8% (74) had HIV RNA less than the lower limit of quantification (<150 copies/ml), and 6% (52) self-reported ever taking antiretroviral therapy (ART). The median degree/egocentric network size was 2 (IQR: 1 to 3). Participants self-reported injecting at 181 unique spatial venues (defined as any public space where two or more PWID reported injecting drugs in the prior 6 months). The network structure and characteristics of participants and injection venues at baseline have been previously described in detail (*32*). All study visits were paused in March 2020 as a result of the SARS-CoV-2 pandemic–associated restrictions.

**Table 1.** Participant baseline characteristics of 2512 PWID in New Delhi, India. Parentheses denote *n* unless otherwise specified. Numbers may not sum to the total if there were participants who elected not to answer a given question.

	**Overall**	**HIV negative at baseline with** **follow-up**	**HIV negative at baseline without** **follow-up**
Number of participants	2512	782	797
Median age (IQR)	26 (22–34)	28 (22–38)	26 (22–35)
Male gender	99% (2489)	99% (771)	99% (789)
Self-report of ever having sex with a man	20% (498)	20% (158)	18% (140)
Self-identify as gay or bisexual	19% (479)	19% (150)	16% (130)
Highest level of education			
No schooling	30% (754)	26% (200)	25% (196)
Primary school (grades 1–5)	25% (618)	22% (169)	24% (190)
Secondary school (grades 6–10) or above	45% (1130)	52% (409)	51% (407)
Employment			
Earn daily wage	62% (1545)	54% (425)	59% (465)
Earn weekly or monthly wage	28% (714)	35% (275)	48% (240)
Unemployed	7% (165)	9% (67)	18% (89)
Currently homeless	30% (754)	21% (160)	26% (210)
Median years injecting drugs (IQR)	5 (2–10)	5 (2–11)	4 (2–8)
Median injections in prior 6 months (IQR)	360 (180–540)	360 (160–540)	360 (144–360)
Mean no. injection partners in prior month	3.2	3.3	2.8
Ever shared syringes	60% (1518)	54% (421)	53% (419)
Shared syringes in prior 6 months	51% (1284)	47% (364)	44% (349)
Type of drug injected (lifetime)			
Heroin only	4% (89)	4% (29)	5% (41)
Buprenorphine only	54% (1350)	53% (413)	62% (491)
Heroin and buprenorphine	42% (1061)	43% (336)	32% (254)
Any other drug only	0% (0)	0% (0)	0% (0)
Type of drug injected (prior 6 months)			
Heroin only	4% (107)	4% (31)	6% (49)
Buprenorphine only	73% (1820)	72% (563)	77% (601)
Heroin and buprenorphine	22% (559)	23% (181)	17% (130)
Any other drug only	0.2% (5)	0.3% (2)	0.4% (3)
Access to services			
Ever tested for HIV	48% (1203)	56% (436)	40% (315)
Ever tested for HCV	4% (104)	6% (44)	2% (17)
Ever used medication-assisted therapy	36% (906)	45% (356)	31% (249)
Ever used syringe service program	17% (427)	19% (147)	11% (87)

By March 2020, all 2512 participants were eligible for at least one follow-up visit. A total of 1266 participants had at least one follow-up before March 2020 (irrespective of HIV status at baseline); 577 participants had one follow-up visit, 393 participants had two follow-up visits, 209 participants had three follow-up visits, and 87 participants had four follow-up visits. A total of 55 participants were confirmed dead. Participants were followed for a median of 12 months, and the median time between follow-up visits was 6 months.

An animation of the evolution of the cohort and network connections over time is available at https://youtu.be/GGoeWTSxmsE. Overall, participants’ injection partner networks (i.e., connections to injection partners) were highly dynamic. Of 1266 participants with at least one follow-up, 75% (947 of 1266) reported a change in injection partners; of these 947 participants, 84% (793 of 947) only reported no longer injecting with a previously named partner, 0.5% (5 of 947) only named at least one new injection partner, and 16% (149 of 947) reported both gaining and losing at least one injection partner. Overall, this equated to an average rate of 1.5 injection partner changes per participant per year, and all newly named partners also reported injecting at the same venue as the participant who named them. Spatial networks (i.e., connections to injection venues) also evolved over time but were more stable than injection networks with 48% (611 of 1266) of participants self-reporting changing injection venues. However, certain injection venues remained common and consistent places where participants injected. The most frequented location (labeled as venue no. 40) was reported at baseline by 48% (609 of 1266) of the participants with at least one follow-up visit. Compared to baseline, an additional 36 participants reported newly injecting at venue no. 40 over follow-up, and 58 participants reported no longer injecting at this venue.

Of 1579 HIV-negative participants at baseline, 782 persons had at least one follow-up visit before the study pause in March 2020. Compared to the 782 HIV-negative participants with at least one follow-up visit, participants without follow-up were significantly more likely to be younger, homeless, report a shorter injection history with less frequent injections in the prior 6 months, and inject heroin only (Table 1). They were also significantly less likely to have access to HIV prevention and testing services. At the network level, participants without follow-up had significantly longer path lengths to a participant with detectable HIV RNA [i.e., the number of persons along the shortest path between a participant (index) and an HIV-detectable participant], reported fewer injection partners (degree centrality), and had lower betweenness centrality. There were no significant differences by needle or syringe sharing, education, or other demographics or risk behaviors.

### HIV incidence

The median age of the 782 HIV-negative persons with at least one follow-up visit was 28 years (IQR: 22 to 38), and 98% (771) were male. All reported injecting drugs in the prior 6 months with a median of 360 injections (two injections per day) (IQR: 160 to 540); 47% (364) reported sharing syringes in the prior 6 months, and 96% (749) reported injecting pharmaceutical opioids, such as buprenorphine (Table 1). As of March 2020, we observed 159 HIV seroconversions over 747 person-years of follow-up, translating to an HIV incidence of 21.3 cases per 100 person-years [Poisson exact 95% confidence interval (CI) = 18.2 to 24.9].

#### Social and spatial network correlates of incident HIV infections

**Fig. 1.**
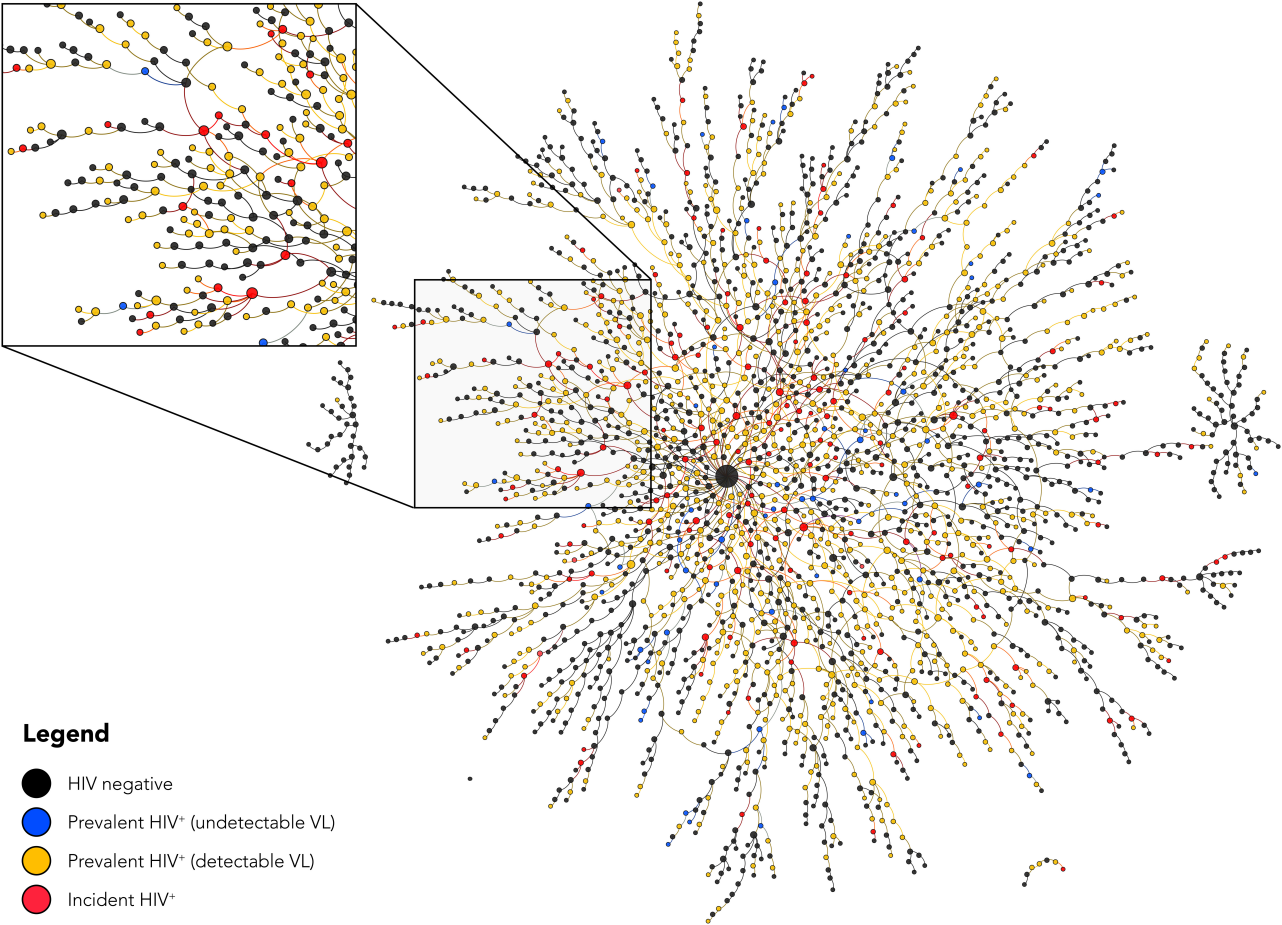
Sociometric injection network of 2512 PWID in New Delhi, India. Nodes represent persons, are sized by degree, and colored by HIV infection status and viral load (VL) as of March 2020; HIV RNA of <150 copies/ml was classified as undetectable. Edges represent an injection partner relationship (i.e., connections to persons with whom a participant injects drugs, irrespective of needle/syringe sharing). Nodes are placed using a degree-dependent force-directed algorithm.

**Fig. 2.**
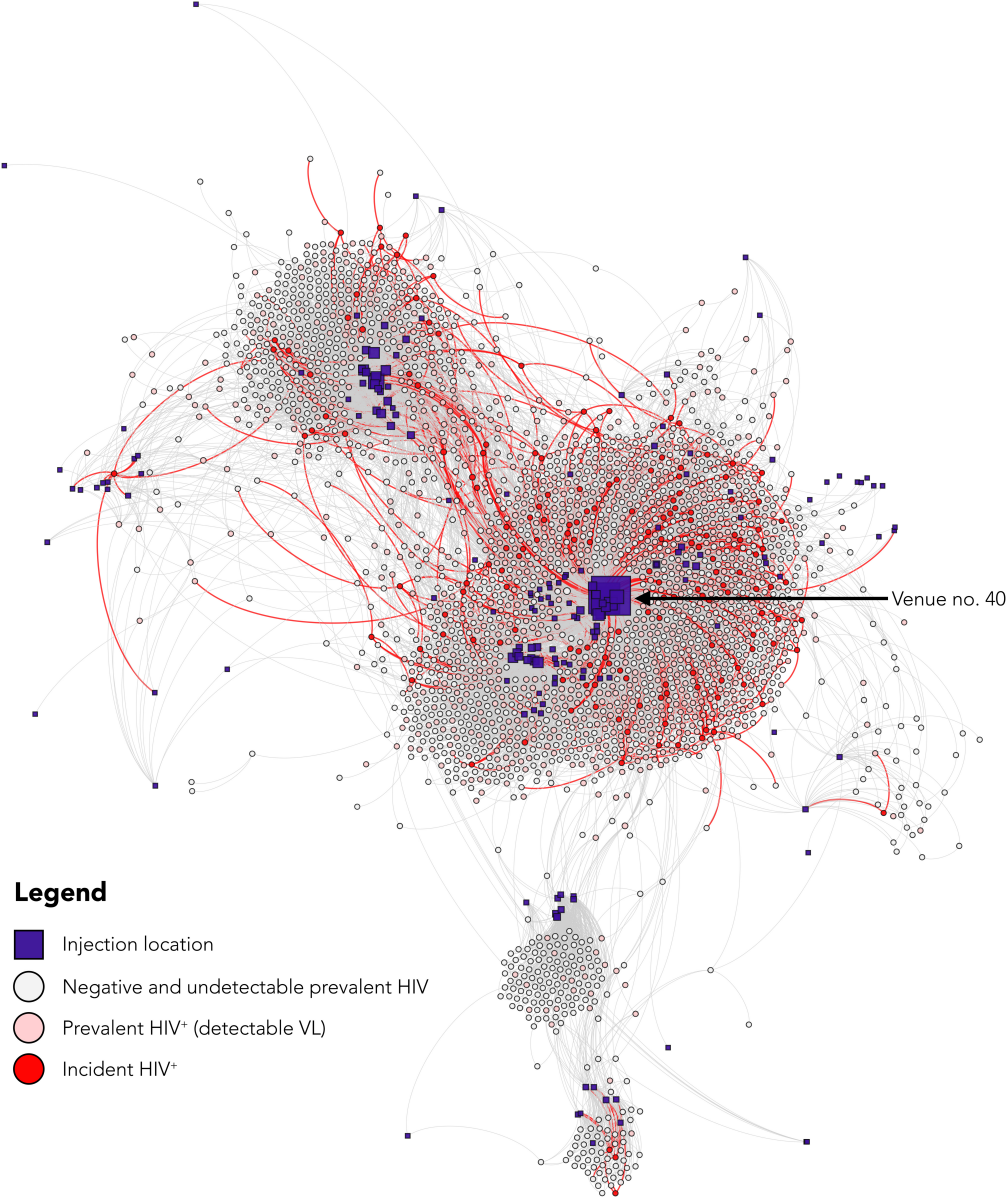
Sociospatial network highlighting HIV incidence among 2512 PWID in New Delhi, India. Nodes represent persons (circles) or injection venues (squares), and edges represent a social tie denoting an injection partner (in the case of a connection between two person nodes) or a spatial tie denoting where a person injects (in the case of a connection between a person node and spatial node). Person nodes are sized by degree and colored by infection status as of March 2020; HIV RNA of <150 copies/ml was classified as undetectable viral load. Red edges show connections with incident HIV infections. Spatial nodes (injection venues) are sized by degree and placed by GPS coordinates to be spatially congruent with their geographic position under a Mercator map projection. Person nodes do not have a geographic location and are placed using a degree-dependent force-directed algorithm that clusters them on the basis of their ties to other nodes.

Of the 159 incident HIV infections, 74% (117) were directly connected to at least one HIV-infected participant with detectable viral load (HIV RNA of >150 copies/ml), and all 159 (100%) incident infections were within one degree of separation from an HIV-detectable participant (Fig. 1). Participants with incident HIV infection reported injecting at 158 unique injection venues with a median of 3 venues per participant (IQR: 2 to 6) before HIV seroconversion. Seventy percent (112) of participants with HIV seroconversion reported injecting at venue no. 40, and, considering indirect ties, 84% (133) were within one degree of separation or less from venue no. 40 (Fig. 2) (an interactive version of this figure is available online at https://sclipman.github.io/SociospatialNet-Incidence/). Betweenness centrality was 2.1 times higher on average among the 159 persons with incident HIV compared to the participants with follow-up who did not seroconvert (two-sample *t* test; *P* < 0.01). In addition, persons with incident HIV infection had significantly higher degree centrality, i.e., number of injection partners compared to those who did not seroconvert (mean degree of 2.6 versus 2.0; *P* < 0.01).

Social and spatial network factors were strongly associated with HIV incidence at multiple levels, even after adjusting for individual-level correlates. At the egocentric level (i.e., direct connections to network members), risk of incident HIV increased by 28% for each additional infected alter with detectable viral load, i.e., HIV RNA of >150 copies/ml [adjusted incidence rate ratio (AIRR) = 1.28; 95% CI = 1.08, 1.50]. At the sociometric level (i.e., one’s indirect ties via alters’ networks), risk of incident HIV decreased by 37% with each additional uninfected participant or participant with undetectable HIV RNA along the shortest path in the injection network separating a given index and a detectable participant (AIRR = 0.63; 95% CI = 0.45, 0.88). Injecting at venue no. 40, an individual’s immediate spatial network was strongly associated with incident HIV infection (AIRR = 3.11; 95% CI = 2.19, 4.42). Last, one’s sociospatial network, capturing indirect spatial ties, was also highly correlated with incident HIV infection even after adjusting for individual- and network-level covariates—risk of HIV acquisition decreased by 26% for each additional person along the shortest path in the injection partner network separating a given participant from injection venue no. 40 (AIRR = 0.74; 95% CI = 0.65, 0.84). All network-level associations were independent of individual-level correlates of HIV incidence that included age, sexual activity, needle sharing, and injection frequency (Table 2).

**Table 2.** Risk factors for HIV incidence by multivariable Poisson regression. Columns represent a regression model and depict the AIRR and 95% CIs for the included variables. An en dash signifies that the variable was not included in the model for that column.

**Factors associated** **with incident HIV**	**Univariable** **model, IRR** **(95% CI)**	**Multivariable** **model 1, AIRR** **(95% CI)**	**Multivariable** **model 2, AIRR** **(95% CI)**	**Multivariable** **model 3, AIRR** **(95% CI)**	**Multivariable** **model 4, AIRR** **(95% CI)**	**Multivariable** **model 5, AIRR** **(95% CI)**
Age (per 5-year increase)	0.80 (0.74–0.88)	0.83 (0.76–0.91)	0.83 (0.75–0.90)	0.82 (0.75–0.90)	0.82 (0.74–0.90)	0.81 (0.74–0.89)
Sexual activity (vaginal or anal sex in prior 6 months)	0.53 (0.38–0.75)	0.62 (0.43–0.88)	0.61 (0.43–0.86)	0.61 (0.43–0.87)	0.69 (0.48–0.98)	0.65 (0.46–0.92)
Shared syringes (prior 6 months)	3.37 (2.35–4.80)	2.50 (1.73–3.63)	2.37 (1.63–3.45)	2.33 (1.61–3.38)	1.99 (1.37–2.88)	2.10 (1.45–3.04)
Injection frequency (per 50 injections in prior 6 months)	1.08 (1.05–1.11)	1.05 (1.02–1.09)	1.05 (1.02–1.08)	1.05 (1.02–1.08)	1.04 (1.01–1.07)	1.04 (1.01–1.07)
Number viremic injection partners	1.31 (1.12–1.54)	–	1.28 (1.08–1.50)	1.03 (0.82–1.30)	–	–
Network distance from an HIV viremic participant	0.61 (0.17–0.79)	–	–	0.63 (0.45–0.88)	0.68 (0.52–0.89)	0.72 (0.55–0.95)
Injecting at a venue no. 40	4.04 (2.88–5.68)	–	–	–	3.11 (2.19–4.42)	–
Network distance from venue no. 40	0.74 (0.63–0.86)	–	–	–	–	0.74 (0.65–0.84)

#### Network modularity and overlapping community detection with GNNs

**Fig. 3.**
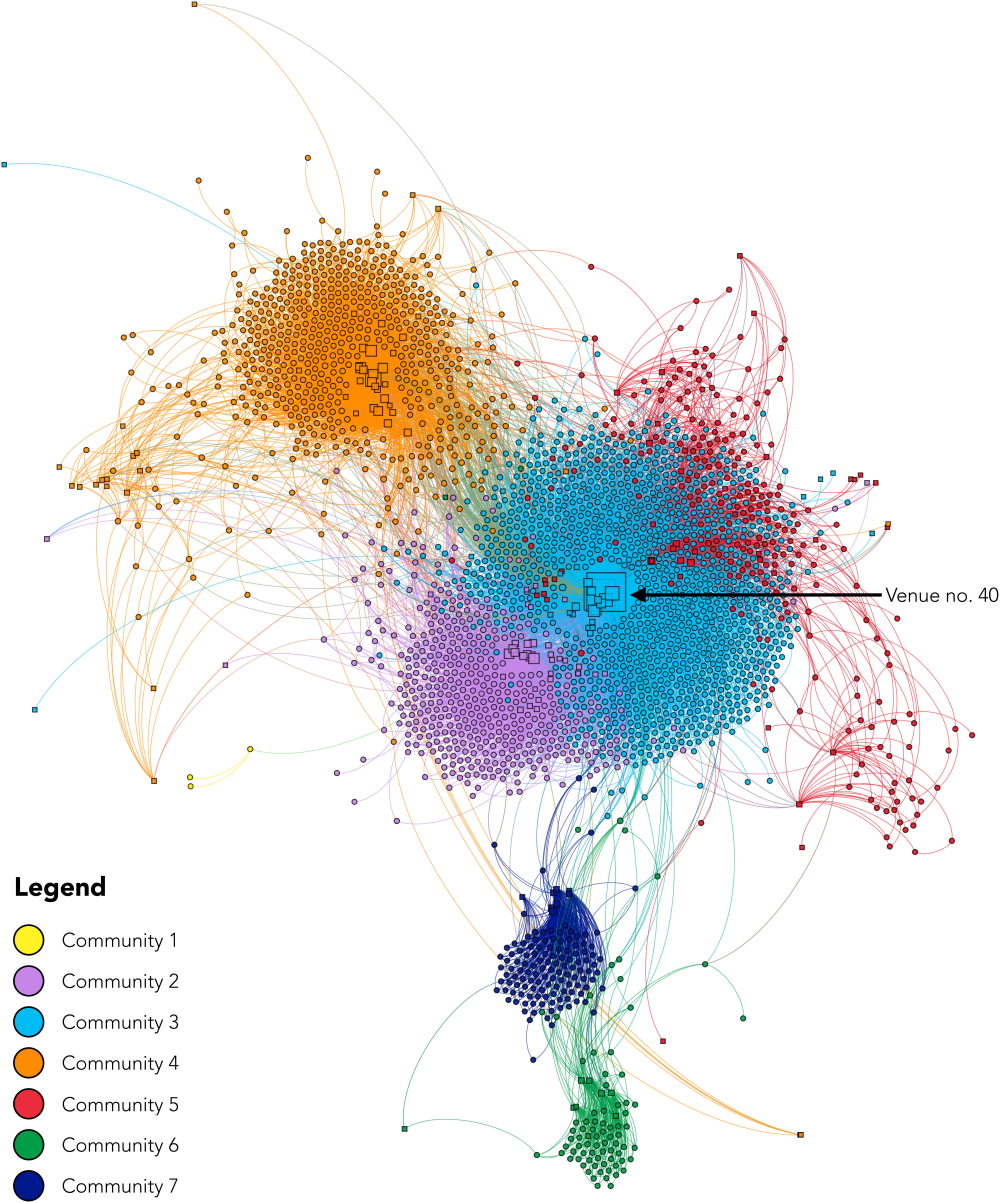
Communities detected by modularity analysis of a sociospatial network of 2512 PWID and 181 injection venues in New Delhi, India. Nodes are sized by degree and colored by community. Circular nodes represent persons, and square nodes represent injection venues (spaces). Spatial nodes are placed by GPS coordinates to be spatially congruent with their geographic position under a Mercator map projection. Person nodes do not have a geographic location and are placed using a degree-dependent force-directed algorithm that clusters them on the basis of their ties to other nodes.

**Fig. 4.**
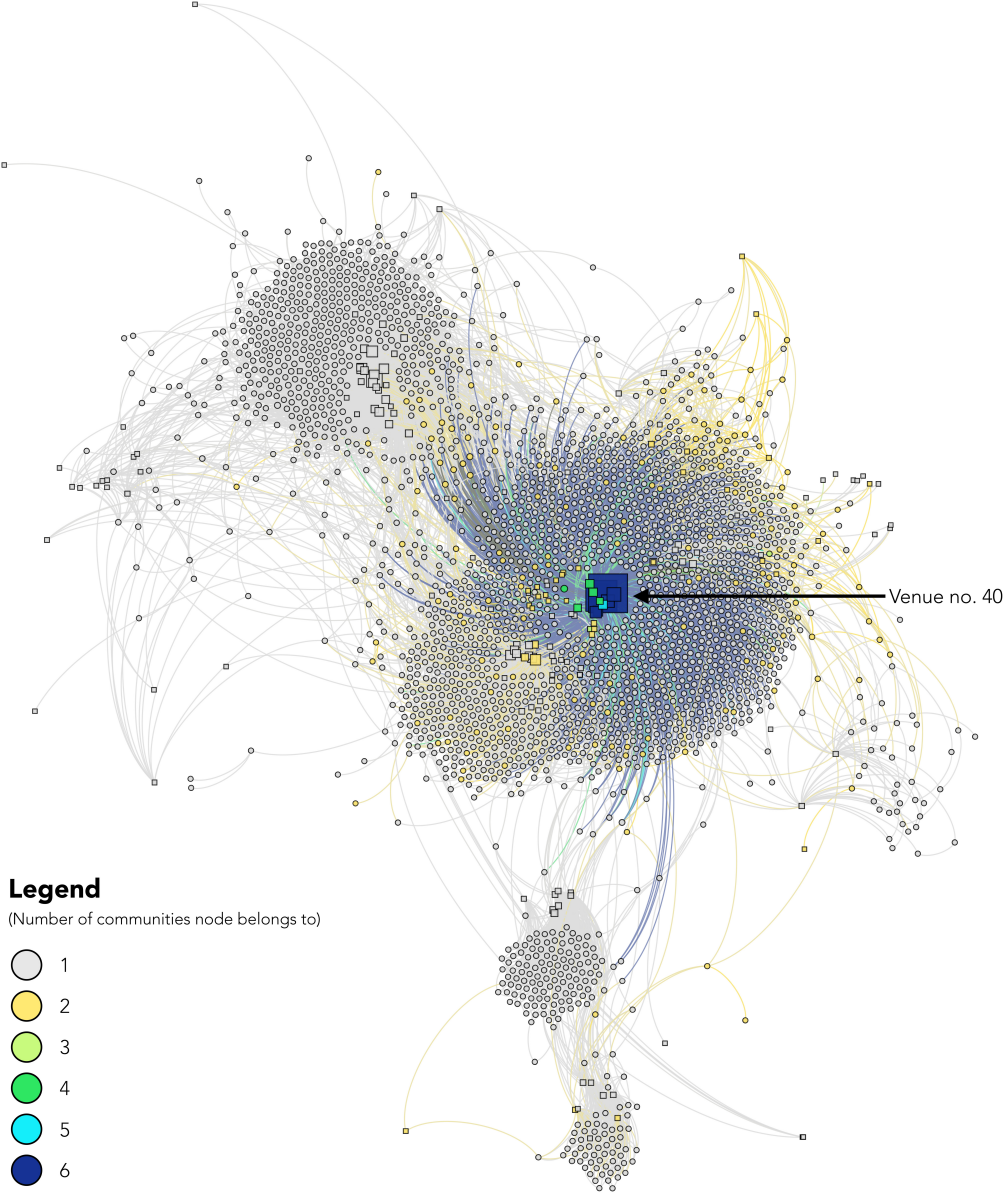
Sociospatial network of 2512 PWID and 181 injection venues in New Delhi, India highlighting the number of communities a node belongs to as identified by NOCD. Circular nodes represent persons, and square nodes represent injection venues (spaces). Nodes are sized by degree and colored by the number of communities the node belongs to. Spatial nodes are placed by GPS coordinates to be spatially congruent with their geographic position under a Mercator map projection. Person nodes do not have a geographic location and are placed using a degree-dependent force-directed algorithm that clusters them on the basis of their ties to other nodes.

Traditional statistical approaches established strong network-level associations with HIV seroconversion and the potential of indirect network ties to be leveraged for the efficient diffusion of interventions. Accordingly, we used community detection and deep learning to identify the nodes (persons or venues) best positioned to reach the greatest number of individuals. First, we used the Louvain method (*33*) to analyze the community structure of the sociospatial network capturing injection partners and the places where PWID inject. Modularity analysis detected seven distinct communities within the sociospatial network, which were primarily geographically localized (Fig. 3); i.e., communities were defined primarily by ties with individuals that inject in similar venues; however, there were significant differences between communities by age, highest level of education, employment status, homelessness, years injected, needle/syringe sharing, and type of drug injected, demonstrating that there is homophily among community members with regard to demographics/risk factors in addition to geography [one-way analysis of variance (ANOVA); all *P* < 0.05]. Incident HIV infections were significantly more likely to cluster within communities, with 69% (109) of incident infections clustering in community no. 3, followed by 18% (*28*) in community no. 4. The highest HIV incidence was observed in community no. 3 (which also contained venue no. 40) at 40.9 cases per 100 person-years (95% CI = 33.9, 49.4) (Table 3). To then determine the extent to which these communities overlap and identify nodes best positioned to maximize reach, we used neural overlapping community detection (NOCD), a deep learning method that combines a GNN with a Bernoulli-Poisson model to detect community overlap (*34*). Overall, we found that 7.5% of nodes in the sociospatial network (i.e., persons and injection venues) belonged to more than one community (Fig. 4). The model showed that select spaces where people inject are most effective at reaching the greatest number of PWID. There were no individuals or injection venues that were shared among all communities; however, eight key nodes overlapped six of the seven communities, all of which were spatial nodes and included the popular injection venue no. 40. Community no. 7 in Fig. 3 was the only community that did not overlap with these key spaces. Person nodes generally belonged to just one community, with 6.0% (150 of 2512) falling into two communities, one person (0.04%) falling into three communities, and one person (0.04%) falling into four communities.

**Table 3.** HIV incidence by network community.

**Community**	**Number of** **incident HIV** **infections**	**Total person** **years**	**HIV incidence** **rate per 100** **person-years** **(95% CI)**
1	0	0	–
2	14	168.25	8.32 (4.93–14.1)
3	109	266.5	40.9 (33.9–49.4)
4	28	244	12.5 (8.60–18.0)
5	5	44.5	11.2 (4.68–27.0)
6	3	19.75	15.2 (4.90–47.1)
7	0	0	–

## DISCUSSION

We observed an exceedingly high HIV incidence in this cohort of PWID in New Delhi, India despite over a third of the cohort being infected at baseline, highlighting ongoing rapid transmission of HIV in this PWID population. The incidence (21.3 per 100 person-years) observed in this cohort between 2017 and 2020 is among the highest incidence rates currently observed in any population globally. Furthermore, these data demonstrate the significant role indirect network connections and space play in fueling the spread of HIV among PWID and the potential application of deep learning on networks to inform infectious disease prevention efforts. Namely, in this sample of 2512 PWID who reported injecting at 181 venues spread over 20 km in New Delhi, NOCD suggested that 95% of the network could be reached by disseminating interventions through any one of eight strategic highly overlapping venues. These interventions could include a wide array of social, behavioral, and pharmacologic approaches, including ART and HIV testing in conjunction with harm reduction strategies such as substance use counseling, syringe service programs, or medication for opioid use disorder.

Over the past two decades, there has been tremendous progress in HIV prevention. With the global scale-up of ART and the introduction of treatment as prevention (TasP) and pre-exposure prophylaxis (PrEP), HIV incidence has plummeted among some of the most vulnerable communities (*35*–*37*). For example, in sub-Saharan Africa where HIV incidence was in the double digits in the early 2000s, it is increasingly being reported that HIV incidence has declined to less than 2.3 per 100 person-years with the scale-up of ART (*35*, *38*–*40*). Similar declines have also been observed among men who have sex with men globally (*41*). However, the HIV epidemic continues to ravage PWID communities despite these advances in HIV prevention and treatment, especially in LMICs (*29*, *31*, *42*–*46*). There are studies from LMICs that report HIV incidence upward of 5 to 10 cases per 100 person-years, but reports of incidence among PWID are scarce (*29*, *31*, *42*–*46*). The incidence observed in this cohort in New Delhi is comparable to a report from Ukraine in 2012 (*42*), which, nearly 10 years later, shows that HIV appears unrelenting in this vulnerable population.

Despite this persistently high incidence, most trials evaluating new pharmaceutical agents for HIV treatment and prevention, such as long-acting ART and long-acting PrEP, have excluded active injectors. Therefore, the expansion of these interventions in PWID populations has been limited. To date, there is only one trial of PrEP among PWID (*47*), the relevance of which to active injectors has been called into question (*48*). Considering the three most recent trials of novel PrEP agents, i.e., DISCOVER, HIV Prevention Trials Network (HPTN) 083, and HPTN 084, the total number of seroconversions observed across ~13,000 high-risk cisgender men and cis- and transgender women was 115 (*35*–*37*). In this cohort in New Delhi, we observed 159 seroconversions among ~800 participants, highlighting the urgent need for the inclusion of PWID populations in HIV prevention and treatment agendas. This need is particularly reflected in settings such as venue no. 40 in this sample. This venue is an open field on the banks of a river in New Delhi where harm reduction services such as syringe service programs and medications for opioid use disorder are within 100 m of the site. However, incidence was highest among participants injecting at this venue, suggesting the need for novel interventions in addition to traditional harm reduction services to curb HIV transmission. Certainly, the incidence observed in this cohort is not unique to New Delhi but potentially PWID populations in several LMICs (*42*)—unfortunately, such data are limited.

Similarly, despite the enthusiasm around the concept of undetectable = untransmissible (U = U), there has been no empirical data to support the construct among PWID. All empirical evidence has come from heterosexual populations and men who have sex with men via their sexual partners (*49*–*51*). Our data reinforce how dynamic PWID network structures make it challenging to evaluate this concept among PWID. However, our network data also provide some support for U = U among PWID. First, 74% of the incident infections were directly connected with at least one participant with detectable HIV RNA. Second, at the egocentric level (direct ties), the risk of HIV incidence increased per alter with detectable HIV RNA. Third, at the sociometric level (indirect ties), the risk of HIV incidence reduced with each additional HIV-negative participant or participant with undetectable HIV RNA between an index and a participant with detectable HIV RNA. We were limited by the lack of phylogenetic information; however, these egocentric and sociometric associations do suggest the potential role of detectable HIV RNA in the transmission of HIV among PWID.

While the individual level and egocentric network associations that we observed are not unexpected, the associations between the empirical spatial and sociospatial network data collected as part of this cohort and HIV incidence are unique, especially from an LMIC setting. While we previously demonstrated (*32*) the strong association of network ties and venues at baseline with HIV and HCV prevalence, here, we advance this concept and provide more justification for venue-based intervention approaches to HIV prevention and treatment. These data suggest a shift in the conceptualization of network structures to transition from purely people to the combination of people and venues. In particular, by leveraging longitudinal as opposed to cross-sectional data, we were able to demonstrate that, while participants’ injection partner networks were highly dynamic, spatial networks were comparatively more stable. Moreover, participants often reported new injection partners from spaces where they commonly injected, reflecting spaces as a more durable point of intervention. The community overlap detection methods further identified that venues, as opposed to individuals, tended to overlap more communities and hence provide a better mechanism for dissemination of interventions.

We posit that the strong associations observed between the spatial and sociospatial network and HIV incidence capture unmeasured connections between PWID injecting at a venue. Most network inventories are challenging to saturate, primarily because of people’s ability to recall—people are more likely to remember injection partners whom they inject with more frequently as opposed to once or twice a month or less frequently. Furthermore, it is possible that participants may have injected with individuals without knowing the persons’ names, thereby limiting their ability to accurately recall and recruit all people with whom they injected. Participants in our study also reported a substantial number of deaths among named injection partners who did not return referral cards; we were only able to confirm a subset of these reports, and there is no central death registry. However, the spatial network, we hypothesize, places all these potential network members within the same network and, consequently, establishes edges between these “undocumented” network participants via the venue. It is likely that the spatial network represents these undocumented connections that arise because of (i) inability of the index to recall all the names of people with whom they injected; (ii) anonymous partners at the injection site whose names the index does not know; and (iii) connections that were not included because a referred network member did not actually return to participate in the study. The risk posed by all these three types of network members, we believe, is captured within the spatial network. A key focus for future research would be to tease out the various contributors to risk in a spatial network. Nonetheless, space as a point of intervention seems more ideal and comprehensive on the basis of these data given these inherent limitations in elucidating social networks.

While such extensive empirical sociometric and spatial network data are rare, the application of novel deep learning methods, specifically GNNs, represents a translational approach to HIV programming. Despite PWID reporting injecting in 181 different injection venues across a diameter of more than 20 km in New Delhi, we detected seven distinct communities of PWID in this sample. Going one step further, NOCD suggested that eight nodes overlapped six of the seven communities, suggesting that these six communities could be reached by rapidly scaling-up services in just one of these eight venues that overlapped these communities (e.g., venue no. 40). In other words, these analyses suggest that the HIV epidemic among this entire sample of PWID in New Delhi could potentially be mitigated by intervening at two strategic points—one venue (e.g., venue no. 40) that overlaps six communities and another venue that reaches the seventh community. If this finding were validated, then it could revolutionize the design of HIV programming to use deep learning methods to target critical points of networks for interventions. For example, saturating these two nodes with harm reduction services, treatment, and PrEP could potentially interrupt HIV transmission among the entire community of PWID in this sample from New Delhi. In addition, given that harm reduction services, including syringe service programs and medications for opioid use disorder, were available at the riskiest venue in this study but were insufficient to curtail transmission, approaches to further tailor these strategies to improve efficiency (e.g., low dead space syringes and more syringes per person) may be warranted.

These results should be interpreted in the context of key limitations. All responses related to drug use, network members, and spaces were self-reported and subject to social desirability and recall bias; to minimize bias, all interviewers were trained on optimal interviewing techniques. About 25% of referral coupons were not returned, suggesting that the networks presented in these data may be incomplete; however, the response rate of 75% is higher than what has been seen in other network studies, and the fact that multiple participants named the same network members suggests high coverage of the network. Furthermore, indexes provided some basic data on network members whose referral cards were not returned and were queried on these nonresponses at the next study visit. One of the main reasons for referral card nonreturn was death of the named member. Among referred partners who were not deceased and did not enroll, all indexes reported injecting with the named partner at a common venue, implying that the missingness would not affect our findings between venue and HIV incidence. It is also possible that some missing participants had detectable viremia, which would strengthen the magnitude of the association with network distance obtained from the Poisson regression. PWID under the age of 18 were excluded from the study because of the legal age of consent in India; therefore, these individuals are not represented by the network topology. As with all network studies, it is possible that participants may not recall or may not list all their network members—we believe the sociospatial network construct addresses some of these undocumented network linkages (*32*). Participants lost to follow-up appear to represent newer and inexperienced PWID with fewer HIV risk factors; however, many new initiates tend to have higher HIV risk because they typically inject with seasoned injectors that have a higher prevalence of HIV. Follow-up in this cohort was paused because of the coronavirus disease 2019 pandemic, and hence, many of these participants did not have an opportunity to complete their follow-up visits. Regressions assumed that observations are conditionally independent on individual-level covariates. This assumption is likely to be violated to some extent, but violations are not expected to bias point estimates (they would result in underestimated SEs). Currently, there are no accepted methods to account for the correlation that arises in sociometric networks. The two methods (*52*, *53*) in the literature that come closest to a valid inference for observational network data are not feasible here, because they are for single time-point data and make strong assumptions about the nature and reach of dependence. NOCD is a novel analytical approach that leverages GNNs to examine community overlap and is yet to be peer-reviewed. Although GNNs are relatively new, studies demonstrating their power and utility are rapidly increasing, and GNNs are being implemented in drug discovery networks and other predictive tasks in medicine (*27*, *54*–*56*). We believe that the NOCD approach to analyzing community overlap offers advantages over existing approaches, such as spectral methods, given its ability to use all available data, including node features. Last, we did not have HIV sequence data available to support linked transmissions from a network member.

Limitations notwithstanding, we documented a high incidence of HIV among PWID in the era of TasP and PrEP, highlighting the need for programs to develop strategies specifically tailored to address HIV among PWID. The strong associations between sociometric and spatial network factors and HIV incidence suggest that it may be pertinent to expand social network studies in the context of infectious diseases to include spaces, and as identified by deep learning methods, venues may serve as the optimal nodes for network-based interventions for maximum efficiency.

## MATERIALS AND METHODS

### Study overview

The Spatial Network Study is an ongoing dynamic longitudinal cohort of PWID in New Delhi, India that was initiated in 2017 (*32*). New Delhi is the capital city of India and is estimated to be home to ~86,000 PWID (*57*)—prior data have shown the HIV prevalence among PWID in New Delhi to range from 13.5 to 35.8% (*29*–*31*). In 2016–2017, cross-sectional HIV incidence (a common tool used in the HIV field that leverages cross-sectional data to estimate incidence based on antibody levels) (*58*) among PWID in New Delhi was estimated to be 18.5 per 100 person-years (*29*). Prior data estimated HCV prevalence among PWID in New Delhi to be 45.7% (*28*). The primary objective of this cohort is to understand the role that different networks play in the transmission dynamics of HIV and HCV among PWID. With the exception of index participants, all participants were recruited via a name generator network referral methodology. Participants completed a baseline assessment and were invited to complete semiannual follow-up visits.

### Study population

The eligibility criteria varied depending on whether the participant was an index or a recruit, as previously described (*32*). Index participants had to (i) be ≥18 years of age, (ii) provide written informed consent, (iii) have self-reported history of injecting drugs for nonmedicinal purposes in the prior 24 months; and (iv) have consented to be recontacted from a prior cross-sectional survey conducted in 2016–2017 (*29*). The eligibility criteria for recruits were as follows: (i) ≥18 years of age, (ii) provide written informed consent, (iii) recruited to participate in the study via a network referral card, (iv) match description provided by their recruiter, and (v) not identified as a duplicate participant by biometric (fingerprint) match. Participants under the age of 18 were excluded because the legal age of consent in India is 18 years. There were no exclusions based on biological sex, gender, or sexual orientation.

### Recruitment

Recruitment of the cohort was initiated with two indexes in November 2017—eight more indexes were included later to account for variability in type of drug injected, marital status, and zip code of residence/injection. As previously described (*32*), all 10 indexes were selected from a cross-sectional sample of PWID in New Delhi accrued for an evaluation assessment of a cluster-randomized trial (ClinicalTrials.gov identifier: NCT01686750) (*29*). When a participant enrolled in the Spatial Network Study, whether the initial 10 indexes or subsequent recruits, they were asked to recall the names of all people with whom they injected in the prior month. In addition, they were asked to provide identifying information about each named network member (e.g., scar on the left hand, one finger missing on the right hand, etc.) and a factoid about each named partner (e.g., “his wife’s name is Priyanka”). Each participant was then provided with a referral card for each named injection partner and was asked to invite them to participate. When these recruits visited the study site, their name and identifying information were compared against the information previously provided. If the information matched, then they were enrolled and asked to name, describe, and recruit people with whom they injected in the prior month (recruit’s egocentric network and the index’s sociometric network). Recruitment continued until the desired sample size (~2500) was reached. Biometric data (fingerprint scans) were used to identify duplicates and establish cross-network linkages (if the same participant was recruited by two different participants). The fingerprint scans were converted to unique hexadecimal codes as previously described (*29*); no images were stored.

### Study procedures

Baseline study visits began with informed consent and referral card validation that included matching the factoid/identifying characteristic provided by the recruiter, followed by biometric registration and identification of duplicates, as previously described (*32*). Participants that were identified as duplicates, i.e., previously enrolled in the cohort, were not enrolled again; however, these data were used to add additional edges (injection partner connections) to the network. At both baseline and follow-up visits, participants completed a survey and blood draw, followed by rapid HIV and HCV antibody testing on-site with appropriate pretest counseling and referrals, as applicable. Participants were provided with referral cards to recruit each of their named injection partners into the study at baseline and follow-up visits if a new injection partner was named. Participants received INR 300 (USD ~4.00) as compensation and could earn an incentive of INR 50 (USD 0.67) per named network partner they referred who was eligible and completed study procedures.

### Data collection

At baseline, participants completed an interviewer-administered electronic survey that captured information on sociodemographics, substance use and risk behavior, sexual risk behaviors and characteristics, social support, quality of life, and access to HIV and HCV services, among others. The survey also captured detailed information about their egocentric injection network, and these data were used to generate referral cards. In addition to injection network data, participants were also asked to list venues where they had injected in the prior 6 months. A list of common injection venues (latitude/longitude) was prepopulated and available on maps of New Delhi to select from. A total of 98 venues were identified through prior ethnography and represented commonly reported injection venues to be included in the survey; however, the survey also allowed for additional sites to be added in real time when reported by participants. Research staff were trained to add locations to the digital maps when participants reported new venues to generate rich spatial data on injection venues. Over the course of the study, 83 additional venues were identified and added to the list by study participants. Once recruitment was initiated, no additional venues were added by the study team; i.e., all new venues that were not originally listed were reported by study participants. Injection venues ranged from public toilets, specific street corners, parking lots, underpasses, graveyards, abandoned buildings/warehouses, to railroad crossings. They were not necessarily shooting galleries or safe injection sites.

At follow-up visits, participants again completed an interviewer-administered electronic survey that updated the information captured at baseline. Participants were also asked whether they were still injecting with the same network members and were asked to name any new injection partners. Similarly, they were asked about places they currently injected at and identify any new venues, if applicable.

### Laboratory procedures

Laboratory procedures at follow-up were the same as baseline study visits (*32*). On-site rapid HIV antibody testing was performed in line with the current standard of care for HIV diagnosis in India using three different kits: Determine HIV-1/2 (Alere Medical, USA), First Response HIV card test 1-2-O (Premier Medical, India), and Signal HIV-1/2 (Arkray Healthcare, India). Rapid HCV antibody testing was performed using the Aspen HCV One Step Test Device (Aspen Diagnostics, India). All residual samples were shipped to the central laboratory in Chennai for RNA quantification and storage. HIV RNA was quantified in all HIV antibody–positive samples using the Abbott HIV-1 RNA RealTime PCR (Abbott Molecular Inc., Des Plaines, IL, USA) with a lower limit of quantification at 150 copies/ml.

### Statistical analysis and computational methods

Statistical analyses were carried out in Python (v3.7.3) and R (v3.5.1). Individual and network variables were analyzed for an association with incident HIV and HCV infection using univariable and multivariable Poisson regression. The Boruta (*59*) random forest feature selection algorithm was used to explore candidate factors. Variables were considered for inclusion in multivariable models if they held biological/epidemiological importance or had significant associations in univariable models or had significant variable importance scores from random forest (*P* < 0.05) (*32*). Sensitivity analyses using less stringent cutoffs (i.e., 0.1, 0.25, and 0.35) were considered to allow for potential covariates to become significant in adjusted analyses and to assess their moderating influence on other variables.

As previously described (*32*), networks were constructed with Python using NetworkX (*60*), and network variables and centrality measures, i.e., degree, betweenness, number of infected injection partners (first-degree alters), and network distance from an infected alter/venue, were calculated from the sociometric network (containing only person nodes). Network distance from an alter with detectable HIV RNA was calculated such that a distance of 0 signifies a direct connection, and a distance of 1 signifies one person who is HIV negative or has undetectable HIV RNA along the shortest path between a given participant and an alter with detectable HIV RNA. Similarly, for network distance from an injection venue, a distance of 0 signifies a direct connection to the venue, and a distance greater than 0 signifies the number of person nodes along the shortest path between a participant and a venue of interest. Networks were visualized using Gephi (*61*), and interactive networks were created using Sigma.js (http://sigmajs.org). Spatial nodes in the network were placed by GPS coordinates to be spatially congruent with their geographic position under a Mercator map projection. Person nodes were placed using a degree-dependent force-directed algorithm.

To analyze community overlap, we first used the Louvain method (*33*) to analyze the community structure of the sociospatial network capturing injection partners and the places where PWID inject. This represents an optimization problem aimed at partitioning the network into communities of densely connected nodes, with the nodes belonging to different communities being only sparsely connected (*62*). The Louvain algorithm is divided into two phases repeated iteratively. It starts with a weighted network of *N* nodes, with each node assigned its own community. Then, for each node *i*, the gain in modularity is calculated when removing *i* from its community and by placing it in the community of neighbor *j* of *i*. Node *i* is then placed in the community that maximizes this gain. The process is applied repeatedly and sequentially for all nodes until there is no further improvement. The method (*33*) computes the gain in modularity Δ*Q* as

$$\Delta Q=[\frac{\sum_{\text{in}}+k_{i,\text{in}}}{2m}-{\left(\frac{\sum_{\text{tot}}+k_{i}}{2m}\right)}^{2}]-[\frac{\sum_{\text{in}}\;}{2m}-{\left(\frac{\sum_{\mathrm{tot}}\;}{2m}\right)}^{2}-{\left(\frac{k_{i}}{2m}\right)}^{2}]$$

where ∑_in_ is the sum of the weights of the edges inside community *C*, ∑_tot_ is the sum of the weights of the edges incident to nodes in *C*, *k_i_* is the sum of the weights of the edges incident to node *i*, *k*_*i*, in_ is the sum of the weights of the edges from *i* to nodes in *C*, and *m* is the sum of the weights of all the edges in the network.

We then used the NOCD, which combines a GNN with a Bernoulli-Poisson model to detect community overlap (*34*). Assume that we are given an undirected graph, represented by a binary adjacency matrix ***A*** ∈ {0,1}^*N* × *N*^, where *N* denotes the number of nodes. Furthermore, each node can be optionally associated with a *D*-dimensional attribute vector, represented as an attribute matrix ***X*** ∈ ℝ^*N* × *D*^. In the absence of node attributes ***X***, we can simply use ***A***. The goal of overlapping community detection is to assign nodes into *C* communities, represented as the community affiliation matrix $F\in R_{\geq 0}^{N\times C}$, where *F_uc_* is the strength of node *u*’s membership in community *c*. This GNN method is described in detail by Shchur and Günnemann (*34*). To recapitulate the method in brief, the Bernoulli-Poisson model is a graph generative model that allows for overlapping communities. Given the affiliations $F\in R_{\geq 0}^{N\times C}$, adjacency matrix entries *A_uv_* are sampled i.i.d. as

$$A_{\mathrm{uv}}∼\text{Bernoulli}(1-\text{exp}(-F_{u}F_{v}^{T}))$$

where ***F****_u_* is the row vector of community affiliations of node *u* (the *u*’s row of the matrix ***F***). The more communities nodes *u* and *v* have in common (i.e., the higher the dot product $F_{u}F_{v}^{T}$ is), the more likely they are to be connected by an edge. The affiliation matrix ***F*** is generated with a GNN

$$F:={\text{GNN}}_{\theta }(A,X)$$

A rectified linear unit (ReLU), a type of activation function used in deep learning models that allows the model to account for nonlinearities and specific interaction effects, is applied elementwise to the output layer to ensure non-negativity of ***F***. The negative log likelihood of the Bernoulli-Poisson model is

$$-\text{log}p(A|F)=-\sum_{(u,v)\in E}\;\text{log}(1-\text{exp}(-F_{u}F_{v}^{T}))+\sum_{(u,v)\notin E}\;F_{u}F_{v}^{T}$$

Because real-world networks are typically very sparse, this equation provides a larger contribution to the loss, which is then counteracted by balancing the two terms, a standard technique in imbalanced classification

$$ℒ(F)=-E_{(u,v)∼P_{E}}[\text{log}(1-\text{exp}(-F_{u}F_{v}^{T}))]+E_{(u,v)∼P_{N}}[F_{u}F_{v}^{T}]$$

where *P*_E_ and *P*_N_ denote uniform distributions over edges and nonedges, respectively. Last, NOCD searches for neural network parameters θ^⋆^ that minimize the (balanced) negative log likelihood

$$\theta^{⋆}=\underset{\theta }{\text{arg}\text{min}}ℒ({\text{GNN}}_{\theta }(A,X))$$

This GNN method has been shown to improve the quality of predictions compared to simpler models (*34*), and while we only considered network connections and did not incorporate individual-level factors (e.g., age, HIV status, and risk behaviors), this approach has the ability to further incorporate node features into the model. The GNN architecture of NOCD uses a two-layer graph convolutional network (GCN) defined as

$$F≔{\text{GCN}}_{\theta }(A,X)=\text{ReLU}(\hat{A}\text{ReLU}(\hat{A}XW^{\left(1\right)})W^{\left(2\right)})$$

where $\hat{A}={\tilde{D}}^{-1/2}\tilde{A}{\tilde{D}}^{-1/2}$ is the normalized adjacency matrix, $\tilde{A}=A+I_{N}$ is the adjacency matrix with self-loops, ${\tilde{D}}_{\mathrm{ii}}=\sum_{j}\;{\tilde{A}}_{\mathrm{ij}}$ is the diagonal degree matrix of $\tilde{A}$, and ***W***^(1)^and ***W***^(2)^ are model weights. NOCD uses a two-layer graph convolutional neural network, with a hidden size of 128, and an output layer of size *C* (number of communities to detect). Batch normalization is applied after the first graph convolution layer. Dropout with 50% keep probability is applied before every layer. A weight decay is added to both weight matrices with regularization strength λ = 10^−2^. The feature matrix ***X*** (or ***A***, in the absence of attributes) is normalized such that every row has unit *L*_2_ norm. For training, the learning rate is set to 10^−3^, and training loss is computed every 50 epochs; optimization is halted if there is no improvement in the loss for the last 500 iterations or after 5000 epochs.

### Ethical clearances

The study protocol was approved by institutional review boards at Johns Hopkins Medicine (IRB00110421) and the YR Gaitonde Centre for AIDS Research and Education in India (YRG292). All participants provided written informed consent.
